# Skin Sympathetic Nerve Activity and the Short-Term QT Interval Variability in Patients With Electrical Storm

**DOI:** 10.3389/fphys.2021.742844

**Published:** 2021-12-22

**Authors:** Songwen Chen, Guannan Meng, Anisiia Doytchinova, Johnson Wong, Susan Straka, Julie Lacy, Xiaochun Li, Peng-Sheng Chen, Thomas H. Everett IV

**Affiliations:** ^1^The Krannert Institute of Cardiology, Indiana University School of Medicine, Indianapolis, IN, United States; ^2^Department of Cardiology, Shanghai General Hospital, School of Medicine, Shanghai Jiao Tong University, Shanghai, China; ^3^Department of Cardiology, Renmin Hospital of Wuhan University, Wuhan, China; ^4^The Division of Cardiovascular Health and Disease, University of Cincinnati, Cincinnati, OH, United States; ^5^Department of Biostatistics, Indiana University School of Medicine & Richard M. Fairbanks School of Public Health, Indianapolis, IN, United States; ^6^Department of Cardiology, Smidt Heart Institute, Cedars-Sinai Medical Center, Los Angeles, CA, United States

**Keywords:** QT interval and corrected QT interval, sympathetic nerve activity, electrical storm, QT interval variability, sudden cardiac death

## Abstract

**Background**: Skin sympathetic nerve activity (SKNA) and QT interval variability are known to be associated with ventricular arrhythmias. However, the relationship between the two remains unclear.

**Objective**: The aim was to test the hypothesis that SKNA bursts are associated with greater short-term variability of the QT interval (STVQT) in patients with electrical storm (ES) or coronary heart disease without arrhythmias (CHD) than in healthy volunteers (HV).

**Methods**: We simultaneously recorded the ECG and SKNA during sinus rhythm in patients with ES (*N* = 10) and CHD (*N* = 8) and during cold-water pressor test in HV (*N* = 12). The QT and QTc intervals were manually marked and calculated within the ECG. The STVQT was calculated and compared to episodes of SKNA burst and non-bursting activity.

**Results**: The SKNA burst threshold for ES and HV was 1.06 ± 1.07 and 1.88 ± 1.09 μV, respectively (*p* = 0.011). During SKNA baseline and burst, the QT/QTc intervals and STVQT for ES and CHD were significantly higher than those of the HV. In all subjects, SKNA bursts were associated with an increased STVQT (from 6.43 ± 2.99 to 9.40 ± 5.12 ms, *p* = 0.002 for ES; from 9.48 ± 4.40 to 12.8 ± 5.26 ms, *p* = 0.016 for CHD; and from 3.81 ± 0.73 to 4.49 ± 1.24 ms, *p* = 0.016 for HV). The magnitude of increased STVQT in ES (3.33 ± 3.06 ms) and CHD (3.34 ± 2.34 ms) was both higher than that of the HV (0.68 ± 0.84 ms, *p* = 0.047 and *p* = 0.020).

**Conclusion**: Compared to non-bursting activity, SKNA bursts were associated with a larger increase in the QTc interval and STVQT in patients with heart disease than in HV.

## Introduction

Sudden cardiac death (SCD) affects over 300,000 people a year in the USA (Myerburg et al., 1997). A major unmet clinical need in cardiology is SCD risk stratification. Abnormal corrected QT interval (QTc) prolongation on the ECG is associated with a threefold increased risk of SCD (Straus et al., 2006). QT variability (QTV) analyses have also been shown to correlate to an increased risk of SCD (Berger et al., 1997; Baumert et al., 2016). We and others have shown that sympathetic tone is a major modifier of both the QT/QTc intervals and QTV (Noda et al., 2002; Piccirillo et al., 2009; Viskin et al., 2010; Page et al., 2016). However, because of technical difficulties, it has been difficult to perform long-term recording of sympathetic nerve activity to determine a direct relationship between sympathetic nerve discharges and QT/QTc intervals and QTV in patients at risk of SCD. We have developed a new method (neuECG) to simultaneously record the ECG and sympathetic nerve activity from the skin by recording signals from ECG electrodes at a high sampling rate and a wide frequency bandwidth (Everett et al., 2017; Kabir et al., 2017; Shen et al., 2017; Uradu et al., 2017; Kusayama et al., 2019). These signals can be differentially filtered to display both ECG (0.5–150 Hz) and skin sympathetic nerve activity (SKNA, 500–1,000 Hz). However, whether or not the SKNA correlates to both the QT/QTc interval and QTV is unknown. We hypothesize that SKNA bursts would be associated with changes in the QT/QTc interval and QTV, especially in patients with heart disease such as electrical storm (ES) and with coronary artery disease but without documented arrhythmias (CHD) which would correlate to an increase in the vulnerability to arrhythmia onset within these patients.

## Materials and Methods

### Study Population

Original data from three patient cohorts, two of which were from a previous study (Doytchinova et al., 2017), were retrieved, and the correlation between SKNA and the QT interval was analyzed. The first group consisted of neuECG recordings from patients admitted with ES (ES group) to the IU Health Methodist Hospital in Indianapolis, Indiana. ES was defined as three or more episodes of sustained ventricular tachycardia and/or ventricular fibrillation over a 24-h period.

The second group consisted of normal healthy volunteers that consented for neuECG recordings during a cold-water pressor test (CPT). The CPT was performed by placing subject’s left hand up to the wrist in iced water for 2 min. A 2-min baseline and recovery period were also recorded before and after CPT.

The third group consisted of neuECG recordings from patients admitted to the IU Health Methodist Hospital that had coronary artery disease (CHD group). This group did not have any documented occurrences of arrhythmias.

All research protocols were approved by the Institutional Review Board of the Indiana University School of Medicine. All cohorts of patients gave written informed consent to participate in the acquisition of neuECG recordings.

### Data Analysis

#### neuECG Recording and Analysis

Signals were recorded as previously described (Uradu et al., 2017; Kusayama et al., 2019). Briefly, neuECG signals which are the simultaneous recording of the ECG and sympathetic nerve activity from the skin consisted of two channels that were recorded with ADInstrument Powerlab. The first channel was an ECG Lead I configuration recorded with one electrode in the right subclavian area and the other electrode in the left subclavian area. The second channel was the ECG Lead II configuration, with one electrode located at right subclavian area and the other in the left lower abdomen. Recordings from both channels were analyzed using the LabChart Pro (ADInstruments). The neuECG signals were sampled at 10 KHz, amplified and bandpass-filtered between 0.5 and 150 Hz to display the ECG and between 500 and 1,000 Hz to show the SKNA. The filtered SKNA signals were then full-wave-rectified and integrated over a 100 ms time window to obtain the integrated SKNA (iSKNA), and the results were displayed over time to simulate the display methods of microneurography. Several hours of recordings were obtained from the ES and CHD groups while they were in the hospital being treated for their conditions.

#### QT Interval and QTV Analysis

**Figure 1:**
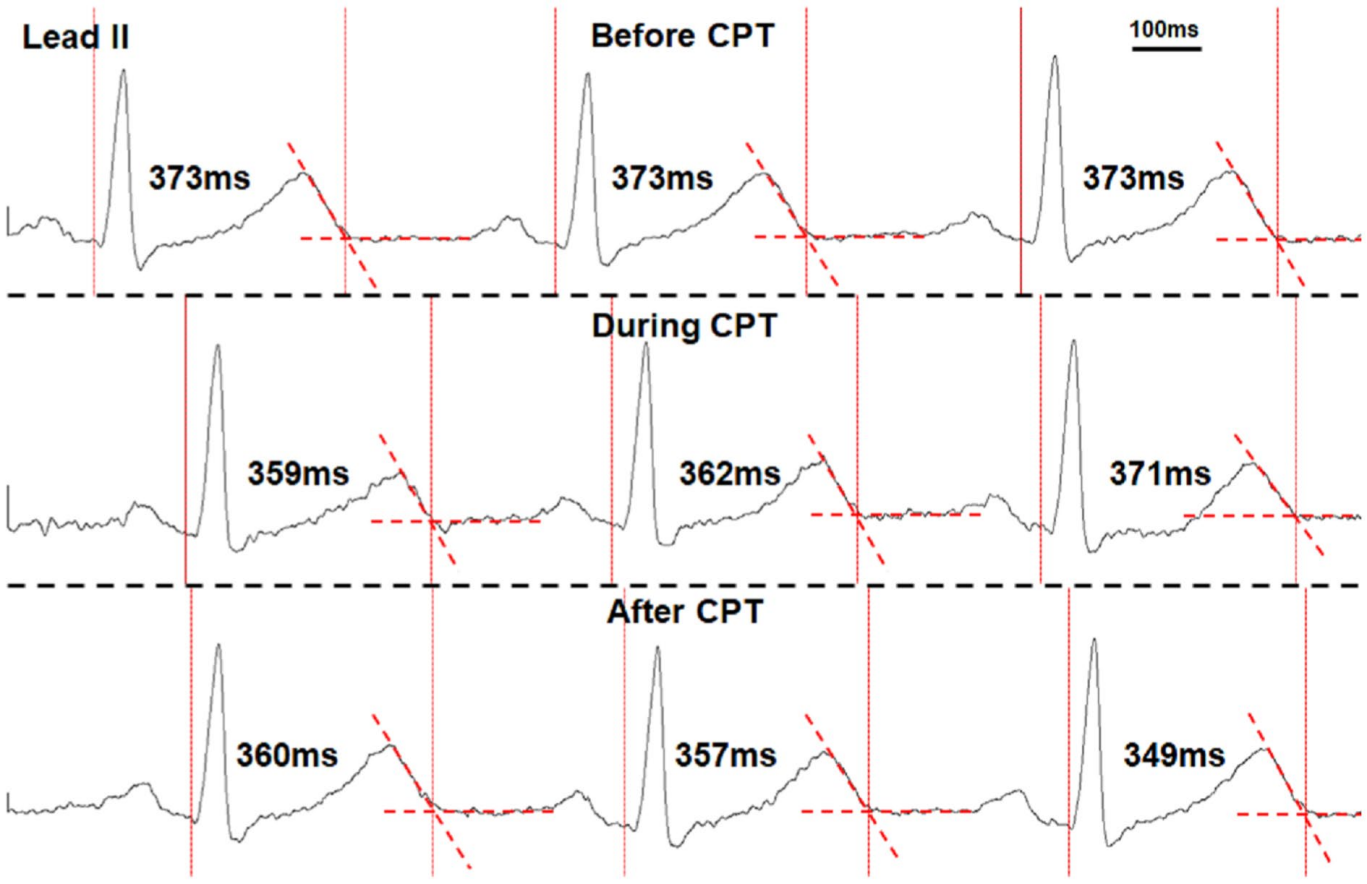
Manual marking of the QT interval using the tangent method in a healthy volunteer performing the cold-water pressor test (CPT). The vertical dotted lines mark the onset of QRS complexes and the end of T waves. The T wave ends when it crosses the isoelectric segments between T and P waves (dashed red line segments). The measured QT interval of each beat is also shown in the figure.

A 6-min ECG recording during sinus rhythm was used for QT interval analysis that was devoid of any arrhythmias and ventricular and atrial premature complexes. QT intervals were manually marked within the Lead II neuECG recording using the tangent method (Postema and Wilde, 2014). Single-beat QT intervals were measured from the beginning of the earliest onset of the QRS complex to the end of the T wave, determined by extending a tangent line from the steepest portion of the downslope of the T wave until it crossed the T-P segment of the signal (Figure 1; Postema and Wilde, 2014). The heart rate was calculated by the Q-Q interval, and QT intervals were corrected for heart rate using Bazett’s formula (QTc; Franz, 1994). The short-term variability of QT interval (STVQT) was calculated every 30 beats for QTV analysis using the following equation: STVQT $=\overset{30}{\underset{n=1}{\sum }}\left|QT_{n+1}-QT_{n}\right|/30\sqrt{2}$ (Baumert et al., 2016). The 30-beat window to calculate the STVQT was then shifted for every subsequent R-R interval, and the STVQT was recalculated. Similarly, the STVQT was calculated in a similar manner for the QTc (STVQTc). These QT interval measures were then compared to both periods of bursting SKNA activity and periods of non-bursting (baseline) activity within the recordings to investigate the correlation between the QT interval and QT variability to SKNA. By comparing the change in QT metrics between the SKNA at baseline with no bursting activity and periods of bursting activity, each patient served as their own control.

#### Burst Analysis of SKNA

**Figure 2:**
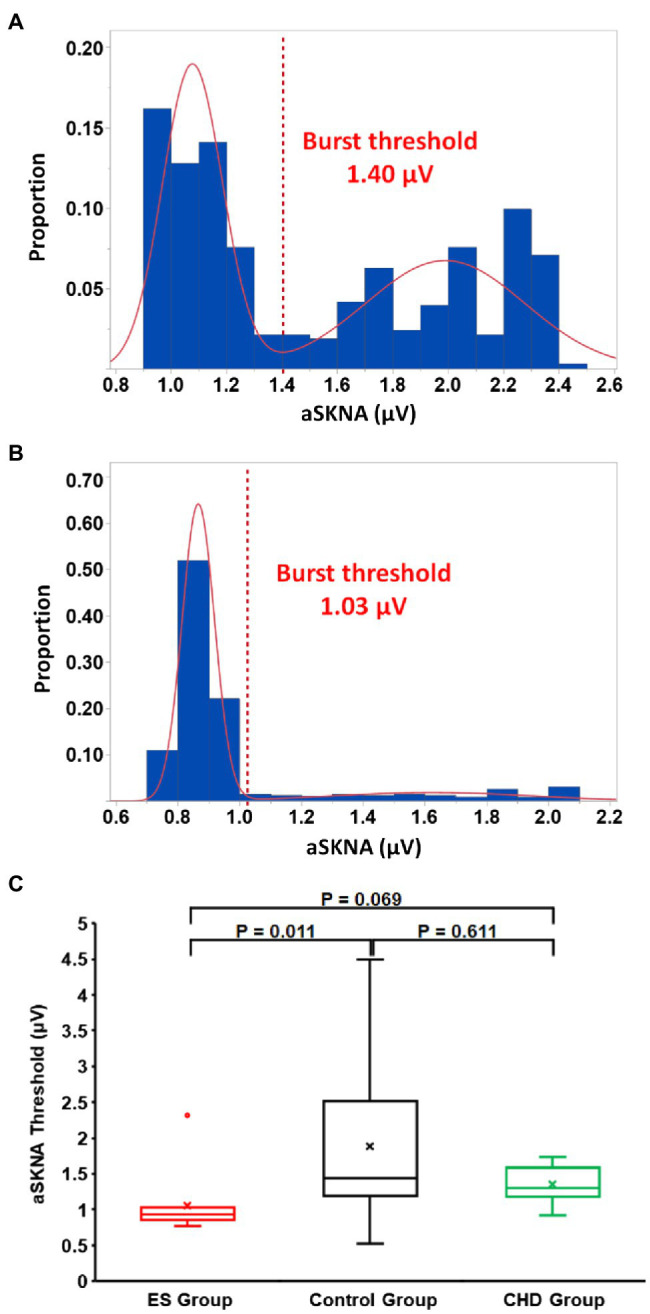
Representative examples of determining skin sympathetic nerve activity (SKNA) burst activity in healthy volunteers (**A**) and ES group (**B**). The SKNA was the averaged SKNA amplitude of 30 consecutive beats. The distribution of SKNA values resulted in two Gaussian distributions. The burst threshold, indicated by a red dotted line, was calculated as the mean representing the lower distribution plus three times the SD. **(A)** The threshold value was 1.40 μV for one healthy volunteer. **(B)** The threshold value was 1.03 μV for one patient with ES. **(C)** Summary threshold data of the three groups. The threshold for a SKNA burst in the ES group was lower than that of control group.

The SKNA amplitude 1-s prior to each QRS complex was averaged to represent the average SKNA (aSKNA) per beat. This averaged SKNA was then compared to the beat-to-beat QT interval. As stated above, the STVQT was calculated over 30 beats. To compare the STVQT to the SKNA, the aSKNA was averaged over those same 30 beats. This calculation also determined for each sliding window of the STVQT calculation such that the timing of the aSKNA and STVQT calculations would be synchronous. Using all aSKNA values, burst analysis was performed as previously described (Kusayama et al., 2019). Briefly, the distribution of the aSKNA values was plotted to visualize baseline (non-bursting) nerve activity and the high amplitude bursting activity. These data were then modeled as two Gaussian distributions. From the Gaussian with smaller mean of the two representing baseline activity, the mean plus three times SD was used as the threshold amplitude for burst determination in each patient (Figure 2).

### Statistical Analysis

Data for all continuous variables are expressed as mean ± SD. Data for categorical variables are expressed as number and percentage. Variables (aSKNA, QT interval, QTc interval, heart rate, and STVQT) are compared among the three groups of CHD, ES, and CPT at the SKNA baseline and the SKNA burst by the Kruskal–Wallis test. If a Kruskal–Wallis test is significant, the Dunn test (1964), a *post hoc* test, is performed to determine which groups differ from each other. The changes of the above variables during the SKNA burst from the SKNA baseline will be compared among groups the same way as above. The changes of the above variables during the SKNA burst from the SKNA baseline within each group are assessed by the Wilcoxon signed-rank test. Multiple comparisons are adjusted for within each outcome variable using the Hochberg method to control the familywise error rate at 0.05.

## Results

### Study Populations

The ES group consisted of 10 patients whose clinical characteristics are shown in Table 1. The CHD group consisted of eight patients with CHD (five males, averaged age 67.3 ± 9.7 years old), and the reason for admittance is listed in Table 2. Another 12 healthy volunteers were enrolled that had interventions known to increase sympathetic tone (five males, averaged age 31.7 ± 7.4 years old). For readability, those variables (aSKNA, QT interval, QTc interval, heart rate, STVQT, and STVQTc) of each group are summarized as Table 3.

**Table 1:** Clinical characteristics of patients with heart disease.

Patient number	ES (*N* = 10)	CHD (*N* = 8)
Age, years old	52.7 ± 12.4	67.3 ± 9.7
Male, *n* (%)	6 (60)	5 (62.5)
Left ventricular ejection fraction, %	38.3 ± 17.6	58.1 ± 17.2
Coronary artery disease, *n* (%)	6 (60)	8 (100)
Non-ischemic cardiomyopathy, *n* (%)	4 (40)	0 (0)
Severe valvular disease, *n* (%)	1 (10)	2 (25)
Heart failure, *n* (%)	7 (70)	1 (12.5)
Beta-blockers, *n* (%)	8 (80)	7 (87.5)
Amiodarone, *n* (%)	2 (20)	2 (25)
Verapamil, *n* (%)	1 (10)	0 (0)

All values are presented as means ± SD or *n* (%).

**Table 2:** Hospitalization of CHD group.

CHD patient	Hospitalization
1	End-stage COPD; underwent bilateral lung transplant
2	David procedure
3	Aortic valve replacement
4	SFA to PT bypass
5	GI bleed
6	Acute hypoxemic respiratory failure
7	Acute kidney injury, ischemic cardiomyopathy
8	Left atrial appendage closure/Watchman procedure

**Table 3:** SKNA and QT interval metrics for each group.

	Healthy volunteers	ES group	CHD group
Baseline			
Duration, s	239.2 ± 71.1	230.7 ± 96.7	218.8 ± 50.6
aSKNA^#^, μV	1.52 ± 0.71^*^^,^^†^	0.89 ± 0.22^*^^,^^†^	1.17 ± 0.20^*^
HR, bpm	70.1 ± 6.1	68.5 ± 11.6^*^	79.7 ± 14.0
QT^#^, ms	364.7 ± 12.3^*^^,^^†^^,^^‡^	482.7 ± 71.1^†^	456.4 ± 88.8^‡^
QTc^#^, ms	392.2 ± 16.4^†^^,^^‡^	511.5 ± 71.9^*^^,^^†^	519.2 ± 84.2^‡^
STVQT^#^, ms	3.81 ± 0.73^*^^,^^‡^	6.43 ± 2.99^*^	9.48 ± 4.40^*^^,^^‡^
STVQTc^#^, ms	6.47 ± 1.46^‡^	7.68 ± 2.87^*^	13.57 ± 5.18^*^^,^^‡^
SKNA threshold^#^, μV	1.88 ± 1.09^†^	1.06 ± 0.45^†^	1.35 ± 0.27
Burst			
Duration, s	108.3 ± 74.5	104.5 ± 65.3	116.0 ± 53.2
aSKNA^#^, μV	2.43 ± 1.42^*^^,^^†^	1.31 ± 0.50^*^^,^^†^	1.54 ± 0.34^*^
HR, bpm	74.2 ± 14.8	70.5 ± 11.0^*^	80.5 ± 13.8
QT^#^, ms	356.3 ± 18.4^*^^,^^†^^,^^‡^	481.2 ± 69.4^†^	456.5 ± 87.5^‡^
QTc^#^, ms	392.4 ± 24.6^†^^,^^‡^	517.2 ± 67.8^*^^,^^†^	522.3 ± 84.0^‡^
STVQT^#^, ms	4.49 ± 1.24^*^^,^^†^^,^^‡^	9.40 ± 5.12^*^^,^^†^	12.81 ± 5.26^*^^,^^‡^
STVQTc^#^, ms	7.27 ± 1.68^‡^	11.60 ± 5.97^*^	17.16 ± 7.61^*^^,^^‡^
Delta change			
aSKNA, μV	0.91 ± 0.90	0.42 ± 0.31	0.37 ± 0.28
HR, bpm	4.1 ± 10.4	2.0 ± 2.8	0.8 ± 1.8
QT, ms	−8.4 ± 14.2	−1.6 ± 8.1	0.1 ± 4.2
QTc, ms	0.2 ± 13.4	5.6 ± 7.7	3.1 ± 5.9
STVQT^#^, ms	0.68 ± 0.84^†^^,^^‡^	2.97 ± 3.06^†^	3.34 ± 2.34^‡^
STVQTc^#^, ms	0.79 ± 1.59	3.92 ± 3.87	3.58 ± 4.03

* *p* < 0.05 when compared between baseline and burst.

# *p* < 0.05 when compared across three groups.

† *p* < 0.05 when compared between the ES group and healthy volunteers.

‡ *p* < 0.05 when compared between the CHD group and healthy volunteers.

### The SKNA and QT Interval in Each Group

**Figure 3:**
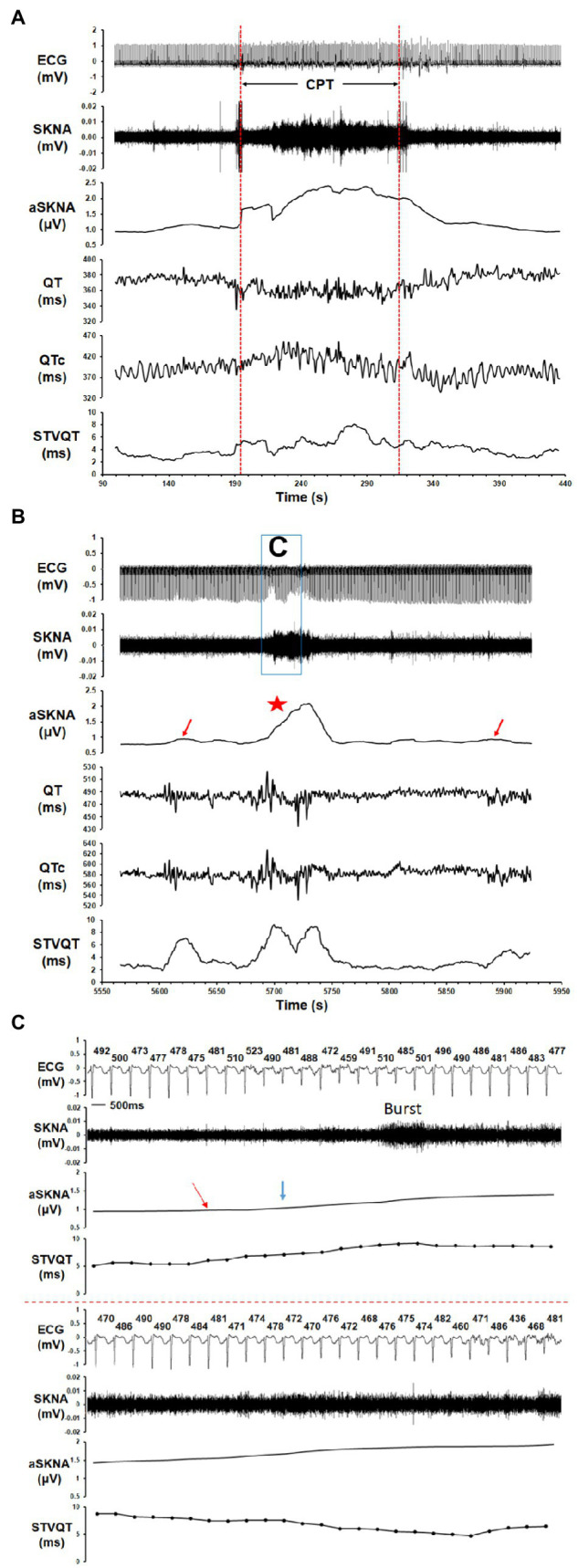
The correlation of SKNA to the QT/QTc interval and short-term variability of the QT interval (STVQT) for healthy volunteers and ES group. Tracings show the ECG, SKNA, average SKNA (aSKNA), QT interval, QTc interval, and STVQT. **(A)** In a healthy volunteer who underwent CPT, the increase in aSKNA correlated to a change in the QT/QTc interval and was associated with an increase in the STVQT. **(B)** In a patient with ES, the aSKNA burst (red star) was associated with an oscillation of the QT/QTc interval and a significantly increased STVQT. Interestingly, the STVQT of the patient with ES increased dramatically even when the aSKNA increased slightly (red arrows did not reach the burst threshold), which may indicate that the vulnerability of STVQT may be influenced by aSKNA in ES patients. **(C)** The example of a SKNA burst and its timing relation to the QT interval and STVQT in an ES patient. The QT interval (ms) of each beat is shown above the ECG tracing. The mean aSKNA at baseline was 0.866. The aSKNA at the red arrow is 0.947. The aSKNA threshold (blue arrow) is 1.025.

**Figure 4:**
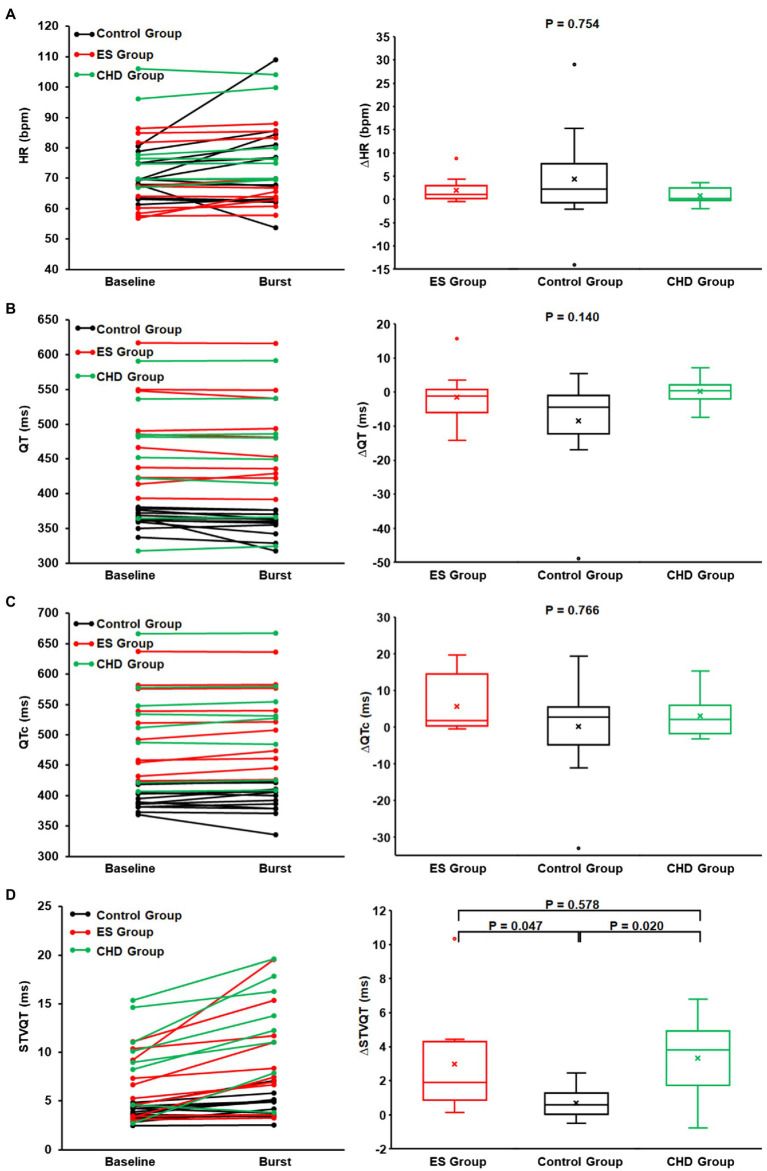
The heart rate **(A)**, QT interval **(B)**, QTc interval **(C)**, STVQT **(D)**, and their differences between SKNA baseline (non-bursting activity) and SKNA burst in healthy volunteers and ES group and coronary artery disease (CHD) group. At SKNA baseline and SKNA burst, the QT interval, QTc interval, and STVQT in ES group (red) and CHD group (green) are significantly higher than those of healthy volunteers (black). Although with higher STVQT at baseline, the STVQT difference between the SKNA burst and baseline in the ES group and CHD group was significantly higher than that in healthy volunteers.

In the healthy volunteers, the threshold to detect burst activity within the SKNA recording was 1.88 ± 1.09 μV (Figure 2C) and the average SKNA amplitude increased from 1.52 ± 0.71 μV at baseline to 2.43 ± 1.42 μV for burst activity (*p* = 0.005). The duration of SKNA burst activity in the healthy volunteers was 108.3 ± 74.5 s (31.0 ± 20.9% of the total duration). At baseline nerve activity levels, the QT interval, QTc interval, heart rate, STVQT, and STVQTc of control group were 364.7 ± 12.3 ms, 392.2 ± 16.4 ms, 70.1 ± 6.1 bpm, 3.81 ± 0.73 ms, and 6.47 ± 1.46 ms, respectively. During burst activity, no significant change was observed in the QTc interval (392.4 ± 24.6 ms, *p* = 0.622), heart rate (74.2 ± 14.8 bpm, *p* = 0.204), and STVQTc (7.27 ± 1.68 ms, *p* = 0.151). However, the STVQT (*p* = 0.016, Figures 3A, 4D) and QT interval (356.3 ± 18.4 ms, *p* = 0.021) was significantly increased during a burst of nerve activity.

In the ES group, the threshold to detect burst activity within the SKNA recording was 1.06 ± 0.45 μV (Figure 2C) and the average SKNA amplitude increased from 0.89 ± 0.22 μV at baseline to 1.31 ± 0.50 μV during burst activity (*p* = 0.002). The duration of SKNA burst activity in the ES group was 104.5 ± 65.3 s (33.3 ± 24.5% of the total duration). At baseline nerve activity, the QT interval, QTc interval, heart rate, STVQT, and STVQTc of ES group were 482.7 ± 71.1 ms, 511.5 ± 71.9 ms, 68.5 ± 11.6 bpm, 6.43 ± 2.99 ms, and 7.68 ± 2.87 ms, respectively. From baseline nerve activity to SKNA burst, no significant change was observed in the QT interval (481.2 ± 69.4 ms, *p* = 0.275). However, from baseline nerve activity to SKNA burst, the QTc interval (517.2 ± 67.8 ms, *p* = 0.010), heart rate (70.5 ± 11.0 bpm, *p* = 0.014), STVQT (9.40 ± 5.12 ms, *p* = 0.002, Figures 3B, 4), and STVQTc (11.60 ± 5.97 ms, *p* = 0.002) were significantly increased.

In the CHD group, the threshold to detect burst activity within the SKNA recording was 1.35 ± 0.27 μV and the average SKNA amplitude increased from 1.17 ± 0.20 μV at baseline to 1.54 ± 0.34 μV during burst activity (*p* = 0.008). The duration of SKNA burst activity in the CHD group was 116.0 ± 53.2 s (34.5 ± 15.7% of the total duration). At baseline nerve activity, the QT interval, QTc interval, heart rate, STVQT, and STVQTc of CHD group were 456.4 ± 88.8 ms, 519.2 ± 84.2 ms, 79.7 ± 14.0 bpm, 9.48 ± 4.40 ms, and 13.57 ± 5.18 ms, respectively. From baseline nerve activity to SKNA burst, no significant change was observed in the QT interval (456.5 ± 87.5 ms, *p* = 0.945), QTc interval (522.3 ± 84.0 ms, *p* = 0.250), and heart rate (80.5 ± 13.8 bpm, *p* = 0.313). However, from baseline nerve activity to SKNA burst, the STVQT and STVQTc were significantly increased to 12.81 ± 5.26 ms (*p* = 0.016) and 17.16 ± 7.61 ms (*p* = 0.039), respectively.

### The Comparison of SKNA and QT Interval Between Groups

No significant difference was found in the duration and proportion of SKNA burst activity across the three groups (*p* = 0.874). No significant difference was found in the heart rate at the SKNA baseline (*p* = 0.091) and SKNA burst (*p* = 0.270) across the three groups. Furthermore, from the baseline nerve activity to burst activity, no significant difference was found in the delta change of aSKNA (*p* = 0.418), heart rate (*p* = 0.754), QT interval (*p* = 0.140), and QTc interval (*p* = 0.766) across the three groups.

During baseline nerve activity, even though the ES group had lower levels of SKNA (*p* = 0.006), the QT interval (*p* < 0.001) and QTc interval (*p* < 0.001) of the ES group were significantly higher than those of healthy volunteers. In addition, even though the ES group had a lower SKNA burst amplitude (*p* = 0.025), the QT interval (*p* < 0.001), QTc interval (*p* < 0.001), and STVQT (*p* = 0.037) of the ES group were significantly higher than that of the control group. The threshold to determine SKNA burst activity within the ES group was lower than that of healthy volunteers (*p* = 0.011, Figure 2C). There was no difference in the SKNA change in magnitude from baseline to burst phase between the two groups (*p* = 0.418). However, from SKNA baseline to SKNA burst, the magnitude of increased STVQT of the ES patients was higher than that of healthy volunteers (*p* = 0.047, Figure 4).

During baseline nerve activity, even though there was the same level of SKNA (*p* = 0.449), the QT interval (*p* = 0.021), QTc interval (*p* < 0.001), STVQT (*p* = 0.011), and STVQTc (*p* = 0.014) of the CHD group were significantly higher than those of the control group (Figure 4). In addition, from SKNA baseline to burst, the QT interval (*p* = 0.011), QTc interval (*p* < 0.001), STVQT (*p* = 0.002), and STVQTc (*p* = 0.004) of the CHD group were significantly higher than those of healthy volunteers (Figure 4). The threshold to determine SKNA burst activity within the CHD group was same as that of healthy volunteers (*p* = 0.611, Figure 2C). There was no difference in the SKNA change in magnitude from baseline to burst phase between the two groups (*p* = 0.418). However, from SKNA baseline to SKNA burst, the magnitude of increased STVQT of the CHD patients was higher than that of healthy volunteers (*p* = 0.020, Figure 4).

During baseline nerve activity, the ES group had the same level of SKNA (*p* = 0.082), and there was no significant difference between the ES group and the CHD group in the QT interval (*p* = 0.332), QTc interval (*p* = 0.952), STVQT (*p* = 0.255, Figure 4), and STVQTc (*p* = 0.102). During SKNA burst activity, there was no significant difference between the ES group and the CHD group in the SKNA amplitude (*p* = 0.207), QT interval (*p* = 0.426), QTc interval (*p* = 0.971), STVQT (*p* = 0.226, Figure 4), and STVQTc (0.215). The threshold to determine SKNA burst activity within the CHD group was same as that of the ES group (*p* = 0.069, Figure 2C). From baseline to burst phase between the ES group and CHD group, there was no difference in the magnitude of changed STVQT (*p* = 0.578, Figure 4) and STVQTc (*p* = 0.890).

## Discussion

The main findings from this study are (Myerburg et al., 1997): Compared to non-bursting activity, the SKNA bursts were significantly associated with an increased QTc interval in the ES group but not in healthy volunteers or the CHD group (Straus et al., 2006). The SKNA bursts were significantly associated with increased the STVQT in all groups (Berger et al., 1997). The SKNA bursts correlated to a greater increase in the STVQT in the ES group and the CHD group than in healthy volunteers.

QT interval variability has been linked to an increased risk of SCD (Berger et al., 1997; Baumert et al., 2016). The data from this study indicate that increased sympathetic nerve activity is associated with increased QTc and STVQT which may increase the vulnerability to ventricular fibrillation within the ES cohort of patients.

The stellate ganglion is a major source of cardiac sympathetic innervation. Studies have shown that sympathetic neurons are located in the skin and have extensive connections back to the stellate ganglion (Taniguchi et al., 1994; Baron et al., 1995). We have shown that SKNA correlates to nerve activity that is recorded from the stellate ganglion (Jiang et al., 2015). These nerve activities occur at frequencies above 500 Hz. The standard high-pass setting for a microneurography study is 700 Hz. By using a bandpass filter from 500 to 1,000 Hz, the ECG components, muscle noise, and environmental noise are eliminated.

### Sympathetic Nerve Activity and the QT Interval

Sympathetic tone is a major factor that controls the cardiac ion channel activity and subsequently the repolarization reserve. Sympathetic nerve activity could potentially either prolong or shorten the action potential duration and QT/QTc interval, depending on the balance between the opposing ionic currents within the existing substrate. In a canine model of heart failure, sympathetic elevation (increased stellate ganglion nerve activity) increases the QT interval (Piccirillo et al., 2009). Another study also showed that nerve growth factor infusion into the left stellate ganglion increases nerve activity and the QTc intervals more than dogs without nerve growth factor infusion (Zhou et al., 2001). These data suggest that increased sympathetic tone and cardiac nerve sprouting is associated with increased QTc interval and the incidence of SCD in dogs with heart failure. Arrhythmogenic ion channel remodeling in heart failure and other heart diseases includes downregulation of K^+^ currents including I_KS_ and upregulation of the I_NCX_, and electrogenic current (Nattel et al., 2007). Therefore, increased sympathetic nerve activity in patients with heart diseases should increase the action potential duration and hence the QTc interval. This supports the data shown in the current study that SKNA burst was associated with an increased QTc interval only in patients with ES but not in the normal healthy volunteers.

A previous study has indicated that changes in autonomic tone, caused by abrupt sympathetic predominance, may cause QTc prolongation in healthy subjects (Andrassy et al., 2007). However, the QTc significantly prolonged at the start of active mental stress (only in the first 10 s), but then normalized by the end of the period (Andrassy et al., 2007). In another previous study, Viskin et al. (2010) found that, in response to brisk standing (i.e., increased sympathetic tone), the QT interval was increased in patients with long QT syndrome but was decreased in controls, and the QTc interval was increased with greater amplitude in patients with long QT syndrome when compared to those controls. Our data showed that the SKNA bursts were significantly associated with an increased QTc interval in the ES group but not in health volunteers, which was consistent with the above-mentioned studies.

### Sympathetic Nerve Activity and QT Variability

Beat-to-beat QTV, an indicator of cardiac electrophysiological stability or the variability of ventricular repolarization duration, has been shown to improve upon the use of the QT interval as a metric for risk stratification for SCD. There are several variables of beat-to-beat QTV, such as variance of QTV, QTV index, and STVQT (Baumert et al., 2016). As QTV is affected by autonomic nerve activity, temporal transition across autonomic states might adversely affect reproducibility in longer recordings (Hnatkova et al., 2013). Due to the transient nature of burst activity within the SKNA recordings, STVQT may be better suited for analyzing the relationship between SKNA and QTV than either the variance of QTV or the QTV index (Feeny et al., 2014; Baumert et al., 2016). In a recent paper, the beat-to-beat variability of ventricular action potential duration, i.e., STVQT, increased during brief periods of increased SKNA in patients with heart failure (Porter et al., 2017). Interestingly, we found that the SKNA bursts were associated with increased STVQT both in patients with ES and in normal healthy volunteers. It is known that sympathetic nerve activity could potentially either prolong or shorten the QT interval. This study has shown that bursts of SKNA lead to changes in the QT interval, augmenting the beat-to-beat difference. As a result, the SKNA bursts were associated with an increase in the STVQT in both groups (Figure 3). Most importantly, the SKNA bursts were associated with increased STVQT to a greater extent in the ES group than in healthy volunteers (Figure 4). The enhanced effect of SKNA bursts on patients with ES than on healthy volunteers could be an effect of a reduction in the repolarization reserve in patients with organic heart diseases (Weiss et al., 2010).

### Clinical Implications

Abnormally increased sympathetic tone is known to be detrimental to cardiac function and plays an important role in the generation and maintenance of cardiac arrhythmias (Everett et al., 2017; Kabir et al., 2017; Shen et al., 2017; Uradu et al., 2017; Zhang et al., 2019). Previous microneurography studies have shown that muscle sympathetic nerve activity (SNA) and skin SNA are independently controlled during heart failure (Middlekauff et al., 1994). Although muscle SNA was markedly increased in heart failure patients, skin SNA was not increased. The actual data show that the heart failure patients had 12 ± 1 skin SNA bursts/min while control had 15 ± 1 skin SNA bursts per min. The small number of patients in that study might have prevented the comparison from reaching statistical significance. Our results are consistent with that prior report. Although SKNA levels (the baseline phase, burst phase and threshold of SKNA) were lower, the QT interval, QTc interval, and STVQT of those patients with ES were higher than those of healthy volunteers. This indicates that SKNA also plays an important role in the occurrence and maintenance of arrhythmias, as the enhanced effect of SKNA on the STVQT was observed in those ES patients (Figures 3, 4). Therefore, the vulnerability of cardiac repolarization (substrate), combined with large SKNA bursts (trigger), would be pro-arrhythmic. Our study provides a new understanding of the effects of sympathetic tone on ventricular repolarization and arrhythmogenesis. Moreover, the changes of QTc intervals and STVQT during SKNA bursts vs. baseline may provide an indication for using SKNA as a novel noninvasive tool for arrhythmia risk stratification. Because repolarization abnormalities occur during the SKNA bursts, these findings also suggest that therapies that suppress SKNA bursts might be useful in SCD prevention and in arrhythmia control.

### Limitations

The number of patients and healthy volunteers enrolled in this study was limited; therefore, the accuracy of the influence of SKNA on QTc interval and STVQT needs further evaluation. The data were obtained while the patients with ES were being managed for their arrhythmias. To compare groups, a 6-min recording was used for SKNA and QT analysis. The QT intervals were manually marked, which may lead to incorrect annotation. However, the QT intervals were marked using the tangent method in order to decrease its measurement error. It is unclear if the antiarrhythmic medications and limited recording time may have affected the SKNA or QT interval analysis. For the QTc interval, only the Bazett’s method showed a difference between the SKNA baseline and burst in the ES group as compared to other QTc calculations. More studies should be performed in the future to test the utility of neuECG for arrhythmia risk stratification.

## Conclusion

Increased sympathetic tone from baseline levels as measured by SKNA bursts was significantly associated with a larger increase in the change of the QTc interval and the STVQT within the ES and CHD patients as compared to healthy volunteers. These changes of the QTc intervals and STVQT during SKNA bursts compared to non-bursting activity indicate that these cohorts of patients may be more susceptible to the onset of ventricular arrhythmias from increased sympathetic nerve activity.

## Data Availability Statement

The raw data supporting the conclusions of this article will be made available by the authors, without undue reservation.

## Ethics Statement

The studies involving human participants were reviewed and approved by Institutional Review Board of the Indiana University School of Medicine. The patients/participants provided their written informed consent to participate in this study.

## Disclosures

Indiana University was awarded U.S. patent No: 10,448,852 for inventing neuECG recording.

## Author Contributions

SC contributed to data analysis and data interpretation and wrote the first draft of the manuscript. GM and JW contributed to data analysis and data interpretation. AD acquired the recordings. SS and JL contributed by enrolling patients in the study, maintaining IRB compliance, and database entry. P-SC and TE contributed to the conception and design of the study, data interpretation, and manuscript editing. XL assisted with the data analysis and data interpretation, contributed to the manuscript revision, read, and approved the submitted version. All authors contributed to manuscript revision, read, and approved the submitted version.

## Funding

This study was supported in part by NIH Grants R41HL124741, R42DA043391, U18TR002208-01, R01 HL139829, OT2OD028190-01, and OT2OD028183-01, the Charles Fisch Cardiovascular Research Award endowed by Suzanne B. Knoebel of the Krannert Institute of Cardiology, a Medtronic-Zipes Endowment, and the Indiana University Health-Indiana University School of Medicine Strategic Research Initiative, and Department of Defense office of the Congressionally Directed Medical Research Programs (CDMRP) under Award Number W81XWH-20-1-0725.

## Conflict of Interest

The authors declare that the research was conducted in the absence of any commercial or financial relationships that could be construed as a potential conflict of interest.

## Publisher’s Note

All claims expressed in this article are solely those of the authors and do not necessarily represent those of their affiliated organizations, or those of the publisher, the editors and the reviewers. Any product that may be evaluated in this article, or claim that may be made by its manufacturer, is not guaranteed or endorsed by the publisher.
